# Improving the time to pain relief in the emergency department through triage nurse-initiated analgesia - a quasi-experimental study from Ethiopia

**DOI:** 10.1016/j.afjem.2024.06.004

**Published:** 2024-06-26

**Authors:** Merahi Kefyalew, Negussie Deyassa, Uqubay Gidey, Maligna Temesgen, Maraki Mehari

**Affiliations:** a Department of Emergency and Critical Care Medicine, Addis Ababa University College of Health Science, Ethiopia; b Epidemiology, Department of Public Health, Addis Ababa University College of Health Science, Ethiopia; c Department of Internal Medicine, Kidus Paulos Specialized Hospital, Ethiopia; d Department of Orthopedic Surgery, Kidus Paulos Specialized Hospital, Ethiopia

**Keywords:** Pain relief, Triage nurse-initiated analgesia, Emergency department, Patient satisfaction, Time to analgesia

## Abstract

**Introduction:**

Pain management is crucial for improving patients' quality of care. Persistent pain has been linked to higher depression, anxiety, and work-related difficulties. This study aimed to enhance the time to pain relief in the emergency department through triage nurse-initiated analgesia. It evaluated the impact of nurse-led analgesia on patient satisfaction compared to standard pain management at Tikur Anbessa Specialized Hospital and Kidus Paulos Specialized Hospital. Additionally, it compared the time to analgesia between the two hospitals and assessed the effect of nurse-led analgesia on reducing the length of stay for patients with pain.

**Methods:**

Using a quasi-experimental design, the study included an intervention group and a control group. Data was collected using an open data kit, and after ensuring data completeness, it was exported to SPSS and Excel for analysis. To assess the effectiveness of the intervention, the time to analgesia was compared between the intervention and control groups using an independent samples *t*-test. This statistical test allowed for a comparison of the mean time to analgesia between the two groups.

Patient satisfaction scores were also compared between the intervention and control groups using the Mann-Whitney U test. Kaplan-Meier curves were employed to compare the time to analgesia between the intervention and control groups within both settings. A point bi-serial correlation analysis was performed to examine the association between the length of stay and the intervention of nurse-led analgesia in both hospital settings.

**Result and discussion:**

The study enrolled 179 participants, with a median age of 34 years (range: 9–80) and 67% female. The most common events leading to pain were medical conditions (21%), followed by trauma/quarrel/war, fall accidents, and underlying diseases (15%, 13%, and 13%, respectively). There was a significant correlation between the degree of pain on arrival and time to analgesia. Additionally, a significant correlation (*p* < 0.01) was found between time to analgesia and patient satisfaction.

**Conclusion and recommendation:**

Implementing a nurse-led analgesia protocol in the emergency department is crucial for reducing time to analgesia and improving patient satisfaction. It is recommended to scale up this approach to other healthcare facilities by incorporating it into the nursing practice guidelines of the country.

## African relevance of this study

− Pain management is a significant challenge in African emergency departments, with limited data available on the prevalence and treatment of pain in this context.
− The study was conducted in two specialized hospitals in Ethiopia, providing important insights into pain management practices in an African setting.
− Addressing the suboptimal pain management practices in African Emergency Departments can improve patient outcomes and quality of care, which is a critical public health issue.
− The findings on the effectiveness of a nurse-led analgesia protocol can inform policy and practice changes to enhance pain relief in African Emergency Departments.
− Scaling up this nurse-led approach to pain management can help address the limited resources and workforce challenges faced by many African healthcare systems.

## Introduction

In 1979, the International Association for the Study of Pain defined pain as "An unpleasant sensory and emotional experience associated with actual or potential tissue damage or described in terms of such damage" [1]. By 2018, this understanding had evolved to encompass six key principles that emphasized pain as a personal experience influenced by biological, psychological, and social factors [2].

Pain is a major concern for patients in the emergency unit [3]. It is estimated that 20% of adults worldwide suffer from pain [4]. In a study conducted in the UK, seven out of every ten patients visiting the emergency department (ED) reported pain as their primary reason for seeking care [5]. Similarly, a study in the USA found that pain assessment was documented for 99.6% of patients upon arrival [6]. In Africa, a pre-hospital study revealed that 27.7% of patients arriving at the ED complained of moderate to severe pain [7]. However, data on in-hospital pain prevalence in Africa, particularly Ethiopia, is lacking.

In Emergency Care, the phenomenon of oligoanalgesia—characterized by inadequate or delayed analgesic therapy—is prevalent [8]. While doctors have faced criticism for not addressing pain effectively, ED nurses often act as strong patient advocates, ensuring that severe discomfort is not overlooked [9]. Various studies have confirmed the significant role of nurses in administering analgesia and sedation [[10], [11], [12]].

Proper pain management is crucial for improving patient care quality. According to the World Health Organization, persistent pain increases the risk of developing depression or anxiety fourfold and doubles the likelihood of work-related challenges [13]. Timely analgesia significantly enhances the quality of care [14]. Despite the high frequency of pain among ED patients, pain management remains suboptimal [15]. An Irish Health Service Executive study found that only 60% of patients in the emergency room with pain sought or received pain relief [16].

Several challenges hinder comprehensive pain control in clinical education. Motov et al. [17] identified several reasons for inadequate pain management in the ED: failure to identify pain, inadequate initial pain evaluation, lack of pain treatment protocols, poor documentation, and failure to meet patient care standards. Administering analgesia after consultation with doctors often delays treatment, diminishing the patient's quality of life [11].

Pain is a common complaint in the ED, with approximately 70% of patients presenting with pain-related issues. Among these, around 40% experience chronic pain [18], while about 45% present with acute pain [19]. In LMIC countries, injuries from road traffic accidents, violence, and self-inflicted harm contribute significantly to disease prevalence among males aged 15–44 [20]. Limited data on pain management exists in Ethiopia, but studies from other African countries indicate high rates of inadequate pain management. For instance, a Nigerian study by Aisouodioneoe et al. [21] found that 45.2% of surgical patients with severe pain did not receive analgesia.

A review by Courtenay et al. [22] of 21 publications on nurse-led care in pain management concluded that educational interventions and specialist nurse protocols effectively reduced pain intensity and were cost-effective. Allione et al. [23] conducted a prospective observational study, finding that 51.3% of patients desired analgesia, but only 72.3% received it. Of those who declined analgesics, the primary reasons were to diagnose pain causes (51.3%) and pain tolerance (44.6%). Van Zanden et al.'s prospective study [24] showed that 43.7% of ED patients desired analgesics upon arrival, while 52.6% refused, with no correlation to demographic or clinical factors.

Timely administration of analgesia often fails to meet patient expectations. Fabbri et al. [25] found that the average time to administer analgesia was 77.6 min. Heilman et al. [26] demonstrated that implementing a standardized care protocol reduced the median time to administer analgesics by 31%. Shahriari et al.'s [27] clinical trial in Iran showed that a pain management program reduced the length of stay (LOS) to 3.2 ± 1.4 days compared to 7.4 ± 4.8 days in the control group. Conversely, Sokoloff et al.'s retrospective study [28] found no significant difference in LOS between patients with and without pain relief.

This study aims to improve the time to pain relief in the ED by implementing a nurse-led analgesia protocol, evaluate its impact on patient satisfaction, compare the time to analgesia between two hospitals, and assess its effect on reducing LOS for patients with pain.

## Methodology

### Study setting

TASH is a prominent healthcare facility with a busy ED that serves approximately 18,000 patients annually, starting from age 13. On average, the ED handles 50 patients daily, including those who are traumatized or critically ill, many of whom require emergency care or resuscitation. The ED is staffed by 14 consultants and 71 residents.

KPH is another key institution, providing healthcare and training through its various biomedical and clinical departments, including Emergency Medicine. The hospital has an inpatient capacity of more than 700 beds and sees an average of 1200 emergency and outpatient clients daily. This extensive patient care load reflects its significant role in both healthcare delivery and medical education. The study was conducted from March 1 to May 31, 2021.

### Study design

The project uses implementation research evaluated by a quasi-experimental design, at the EDs of TASH and KPH. The intervention arm received an intensive program of nurse-led analgesia, the control ED received routine pain management for patients in need. The evaluation had a baseline data collection followed by an intervention. Finally, at the discharge of patients, the study had a post-intervention end-line survey. The baseline data was essential to assess the socio-demographics and conditions related to the immediate causes of the emergency. At the same time, the post-intervention end-line survey was used to determine the impact of the intervention. To assess the knowledge and practice of pain assessment and management among nurses working in the Triage department, a standardized tool was developed based on a previously published research tool in Gondar [29]. Out of the total of 59 ED nurses, 10 willingly participated in the pre-training test by completing the questionnaire. The nurses who were not willing to participate or those working in departments other than Triage within the ED were not included in the study. The mode of delivery of the training used an interactive illustrated lecture. The main foci of the training was assessment of life-threatening conditions, assessment of NRS of the patient's pain, and evaluation of the effects of analgesics. Ten experts were involved to assess its content validity, and it was 0.8. In response to the identified knowledge gap, a training module was developed, and the participants received interactive training over two days. This training was specifically designed to enhance their knowledge and was conducted by both the Principal Investigator (PI) and the co-investigator. Following the two-day training, a post-training test was administered, and only individuals who achieved a score of 70% or higher were eligible for inclusion in the study.

To assess the shortening in the LOS of patients with pain, the study used sample size determination for the mean difference in the two populations. The study used taking Z_α/2_ with a critical value of the normal distribution at α/2 with a confidence level of 95%, (1.96), Z_β_ is the crucial value of the normal distribution at β with a power of 80%, (0.84), and a mean difference between the two groups of 5 min, considering 77.6 min as in a study done by Tanabe et al. [15] with a standard deviation of 20 and 25 min in the two arms with a ratio of one to one in each group. Based on the above assumption, the study needed 322 from each component, adding 10% for non-response or incompleteness; the research required 355 from TASH, and another 355 patients with pain at admission from KPSH.

### Data collection

The study determined a sample size of 218 participants for each group, calculated using the double proportion population formula. The intervention group was recruited from patients at TASH who met the inclusion criteria, while the control group was recruited from patients at KPH who fulfilled the same criteria.

A questionnaire was developed for data collection, covering socio-demographic characteristics, arrival time at the ED, pain assessment details (such as onset time, severity, transportation method, pre-medication measures, etc.), medication provided, time of pain relief, and overall satisfaction level. The questionnaire underwent pre-testing, and necessary corrections were made based on the feedback received. The interviews were conducted using Open Data Kit (ODK) on Android mobile/tablet devices. Numeric scales were provided on paper for patients to indicate their pain severity and satisfaction levels accurately.

Experienced data collectors, trained in interviewing methods and familiar with the questionnaire content, conducted the interviews. If the patient was not in a condition to respond, a caregiver was interviewed, except for pain-related questions and the final satisfaction question.

Data quality was ensured through the use of a pre-tested questionnaire and trained data collectors. The ODK data collection instrument was programmed to minimize missing entries, ensure proper skipping of questions, and limit data entry errors. The data collectors were supervised by co-investigators who work in the ED. Data collected through the interviews using ODK on Android smartphones was coded and subsequently exported to Excel and SPSS for Windows version 26 for further analysis.

### Data analysis

Descriptive statistics were used to summarize the socio-demographic characteristics of the study participants. Frequencies and percentages were calculated for categorical variables such as gender, age, residence, marital status, education level, referral mode, payment method, and occupation. The type of problems patients had was presented as frequencies and percentages.

To assess the effectiveness of the intervention, the time to analgesia was compared between the intervention and control groups using an independent samples *t*-test. Patient satisfaction scores were also compared between the intervention and control groups using the Mann-Whitney U test. Further analysis was conducted on patients who were discharged from the ED and those admitted to the inpatient ward. Kaplan-Meier curves were used to compare the time to analgesia between the intervention and control groups in both settings. A point bi-serial correlation analysis was performed to examine the association between the LOS and the intervention of nurse-led analgesia in the two hospital settings. A P-value of <0.05 was considered significant.

## Results

One hundred seventy-nine participants were enrolled in this study, and their median age was 34 (9–80) years. The socio-demographic characteristics of the participants in the intervention and control group are revised in the table below. (Table 1)

**Table 1:** Socio-demographic characteristics of the participant.

**Socio-demographic variable**	**Sub-variable**	**Frequency**		**Total**	
		**TASH**	**KPSH**		
**Gender**	Male	30	27	57	
	Female	60	62	122	
**Age (years)**	<21	8	8	16	
	21–30	27	29	56	
	31–40	22	18	40	
	41–50	12	12	24	
	51–60	8	9	17	
	60+	13	13	26	
**Residence**	Addis Ababa	48	50	98	
	Out of Addis Ababa	41	40	81	
**Marital status**	Married	60	51	111	
	Never married	24	24	48	
**Education level**	No formal education	21	23	44	
	Primary school	29	30	59	
	Secondary school	23	22	45	
	Higher school	16	15	31	
**Referral mode**	Self from home	30	32	62	
	central triage	2	0	2	
	OPD	11	11	22	
	Addis Ababa public hospital	9	7	16	
	regional public hospital	21	20	41	
	health center	16	17	33	
	private hospital	1	2	3	
**Payment method**	Paying	65	64	129	
	Free	25	25	50	
**Occupation**	Health professional	1	1	2	
	Driver	4	5	9	
	Bank/supermarket/shop worker	4	4	8	
	Daily labourer/ petty trading	16	16	32	
	Stable services (Governmental/NGO)	18	22	40	
	Work at home	27	23	50	
	Student	13	13	26	
	Others	6	6	12	

TASH= Tikur Anbessa Specialized Hospital, KPH= Kidus Paulos Specialized Hospital.

**Fig. 1:**
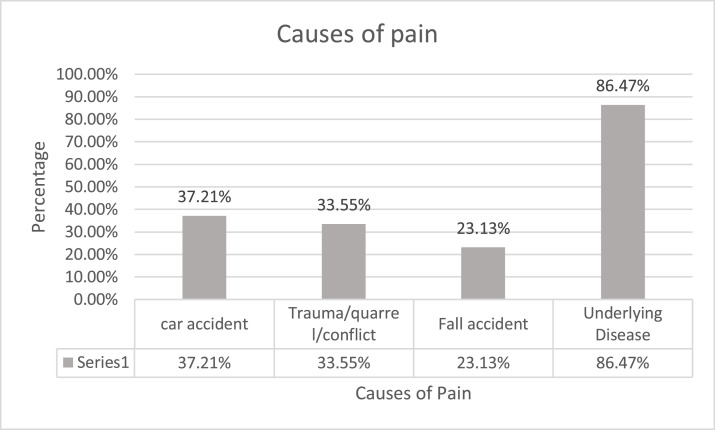
Types of problem* others include Burns, cancer, Acute abdomen, DVT, renal stone, back pain and others Complex trauma is exposure to varied and multiple traumatic events.

With regards to the type of problems patients had, road traffic accident (RTA) is the most common event, accounting for 37 (21%). The rest of the findings are shown in Fig. 1 below.

The average time to analgesia in the intervention and control group were 88.9 and 419.9 min, respectively. The intervention group showed a statistically significant reduction in the time to analgesia (*p* < 0.01). This suggests that patients in the intervention group experienced a shorter time to receive analgesia compared to the control group (Table 2).

**Table 2:** Independent samples *t*-test to compare the time to analgesia and patient satisfaction between the intervention and control groups.

Variable	Variables				
	t-value	Degree of freedom(df)	Significance level(P-value)	Mean difference	95% of confidence interval of the difference
Time to analgesia	−5.654	171	<0.01	−331.09	−446.69 - −215.5

The Mann-Whitney U value is 3326.500, and the Wilcoxon W value is 7421.500. The Z value is −2.056, and the Asymptotic Significance (2-tailed) is 0.04. The Asymptotic Significance indicates that the difference in satisfaction between the two groups is statistically significant. with the Intervention group reporting higher satisfaction.

**Fig. 2:**
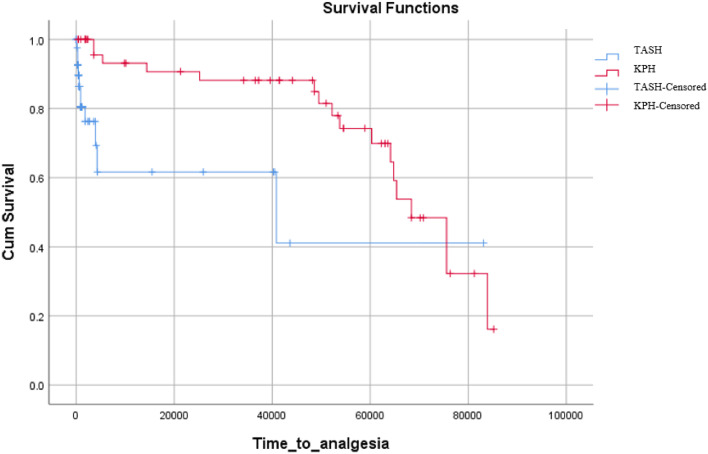
Kaplan Meir Curve showing time to analgesia of patients of TASH and KPH TASH= Tikur Anbessa Specialized Hospital, KPH= Kidus Paulos Specialized Hospital.

Among patients who were discharged from the ED, there was a statistically significant difference (*p* = 0.01) in the time to analgesia between the intervention and routine care groups. This suggests that patients in the intervention group had a significant shorter time to receive analgesia compared to the control group. It is depicted in Fig. 2.

**Fig. 3:**
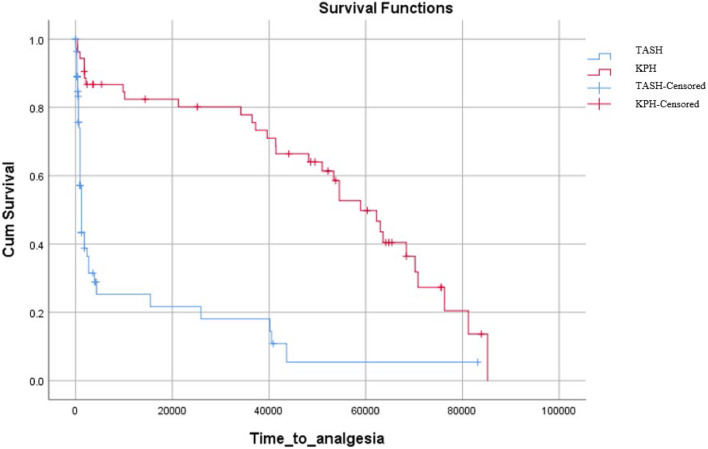
Kaplan Meir Curve showing time to analgesia of In-patient ward admitted patients of TASH and KPH. TASH= Tikur Anbessa Specialized Hospital, KPH= Kidus Paulos Hospital.

In patients admitted to the in-patient ward, there was a statistically significant difference (*p* < 0.01) in the time to analgesia between the intervention and control groups. This implies that patients in the intervention group experienced a significantly shorter time to receive analgesia compared to the control group.(Fig. 3).

The point bi-serial correlation analysis indicated that there was no statistically significant association (p-value = 0.294) between the LOS and the intervention of nurse-led analgesia in the two hospital settings.

## Discussion

This study revealed that trauma-related causes accounted for approximately one-third of the cases, which is consistent with previous literature conducted in Africa by Colin et al. [20]. However, the average time to analgesia in this study was longer compared to the study conducted by Fabbri et al. Andrea and colleagues (88.9 min vs. 77.6 min). Nonetheless, considering the skewed distribution of the data, the median with interquartile range (15 and IQR=15) was significantly lower than the findings reported by Andrea et al. and Tenabe et al. [[15], [25]].

Similar to the findings of Goh et al. [11], time to analgesia is significantly delayed in routine care compared to nurse-led analgesia.

Moreover, the study showed a significant correlation between time to analgesia and patient satisfaction. This finding aligns with the literature that emphasizes the importance of timely pain relief in improving patient outcomes and satisfaction. The study by Heilman et al. [26], which focused on reducing pain relief time for ED patients with extremity fractures, also demonstrated the positive impact of implementing standardized care protocols on reducing the time to analgesia. Similarly, Magner et al. [10] highlighted the benefits of nurse-led analgesia in improving the utilization of sedatives and analgesics in ventilated infants after cardiac surgery.

This study builds on prior research, finding that a nurse-led analgesia protocol reduced time to pain relief and improved patient satisfaction. This aligns with the work of Courtenay et al. [22], which has also demonstrated the benefits of this approach in expediting pain management. Providing prompt pain relief is crucial, as delays can lead to negative consequences like increased complications and prolonged patient suffering.

Prompt analgesia is linked to higher patient satisfaction, as it improves physical comfort and the overall patient experience.

## Conclusion and recommendation

The findings of this study support the implementation of nurse-led analgesia protocols in the ED, which has demonstrated a positive impact on reducing the time to pain relief and improving patient satisfaction. By emphasizing the crucial role of nurses in pain management and implementing standardized care protocols, healthcare facilities can effectively address pain in the ED and enhance patient outcomes.

Based on the results of this study, it is recommended to scale up the nurse-led analgesia approaches in other healthcare facilities.

### Limitation

Conducting a study in a busy ED setting presents unique challenges, primarily due to the high demand for immediate and critical patient care. Healthcare providers in these settings are often faced with time constraints as they prioritize attending to patients in urgent need of medical attention. This fast-paced environment makes it difficult to allocate sufficient time and resources for recruitment activities, such as approaching potential participants, explaining the study details, and obtaining informed consent.

### Dissemination

Result from this study was shared with staff members at the data collection site through an informal presentation. The results were also presented on the annual research day in the institution where intervention arm was applied.

## Declaration of competing interest

The authors declare no conflicts of interest that could potentially bias or influence the outcome of this study. Funding for the project was provided by the Zoll Foundation and the Health Professionals Education Partnership Initiative (HEPI) Ethiopia, but these funding sources had no involvement in the study design, data collection, analysis, interpretation, or the decision to publish the findings. The authors have adhered to ethical guidelines and scientific integrity throughout the research process, ensuring transparency and objectivity in reporting the results.
